# Comment on “A 10‐Year Longitudinal Study of Muscle Morphology and Performance in Masters Sprinters” by Hendrickse et al.

**DOI:** 10.1002/jcsm.13857

**Published:** 2025-06-04

**Authors:** Yu‐Hsiang Lin, Jau‐Yuan Chen, Kuo‐Jen Lin

**Affiliations:** ^1^ Department of Urology Linkou Chang Gung Memorial Hospital, Chang Gung University School of Medicine Taoyuan Taiwan; ^2^ Department of Family Medicine Linkou Chang Gung Memorial Hospital, and School of Medicine, Chang Gung University Taoyuan Taiwan

We read with great interest the recent longitudinal study by Hendrickse et al., which examined changes in muscle morphology and performance in Masters sprinters over a 10‐year period [1]. The authors found preserved muscle histology yet a significant decline in functional outcomes such as sprint time and jump power. While this is an important contribution to our understanding of ageing muscle, we would like to offer a complementary perspective on the role of mitochondrial energetics and systemic hormonal factors that may underlie these functional declines.

It is increasingly recognized that age‐related muscle dysfunction is not solely a matter of muscle mass or fibre morphology but is also strongly linked to mitochondrial capacity and bioenergetic efficiency. Several studies have shown that mitochondrial function directly correlates with muscle strength, power output and mobility in older adults [2, 3, 4]. Grevendonk et al. demonstrated that even physically active older individuals experience reduced mitochondrial oxidative capacity, and only sustained high‐level training can significantly preserve mitochondrial health and physical function [3].

Moreover, ageing is often accompanied by sleep disruption due to conditions such as benign prostatic hyperplasia (BPH) and obstructive sleep apnea (OSA), both of which contribute to testosterone decline via circadian and neuroendocrine disturbances [5, 6]. Even short‐term sleep restriction has been shown to significantly reduce testosterone levels in healthy young men [7]. This decline in testosterone, in turn, has been mechanistically linked to increased oxidative stress and mitochondrial dysfunction, further impairing muscle bioenergetics [8].

Lin et al. recently proposed a comprehensive model linking ageing, sleep dysregulation, hormonal axes (HPG, HPA and HPT) and mitochondrial failure to the pathogenesis of metabolic and neurodegenerative diseases [9]. Such hormonal‐mitochondrial axes may also play a central role in the loss of physical performance observed in ageing athletes, even in the presence of preserved muscle histology.

Additionally, we highlight that mitochondrial dysfunction is not restricted to skeletal muscle. Zhou and Tian have detailed how mitochondrial failure in the ageing heart compromises energy delivery to peripheral tissues, including muscle, contributing to exercise intolerance and functional loss [10].

In summary, we commend the authors for their rigorous long‐term study and propose that future research include assessments of mitochondrial function, hormonal status and sleep quality to more fully elucidate the multifactorial mechanisms underlying physical performance decline in ageing populations.

## Conflicts of Interest

The authors declare no conflicts of interest.

## Declaration of Generative AI and AI‐Assisted Technologies in the Writing Process

During the preparation of this work, the author(s) used ChatGPT in order to refine the manuscript and enhance the fluency of the English language. After using this tool/service, the author(s) reviewed and edited the content as needed and take(s) full responsibility for the content of the publication.

## Data Availability

No new data were generated or analysed in support of this work.
